# Association of psychosocial factors at work with fertility and menstrual disorders: A systematic review

**DOI:** 10.1111/jjns.12624

**Published:** 2024-10-17

**Authors:** Natsu Sasaki, Kotaro Imamura, Kazuhiro Watanabe, Yui Hidaka, Asuka Sakuraya, Emiko Ando, Hisashi Eguchi, Akiomi Inoue, Kanami Tsuno, Yu Komase, Mako Iida, Yasumasa Otsuka, Mai Iwanaga, Yuka Kobayashi, Reiko Inoue, Akihito Shimazu, Akizumi Tsutsumi, Norito Kawakami

**Affiliations:** ^1^ Department of Mental Health, Graduate School of Medicine The University of Tokyo Tokyo Japan; ^2^ Department of Digital Mental Health, Graduate School of Medicine The University of Tokyo Tokyo Japan; ^3^ Department of Public Health Kitasato University School of Medicine Sagamihara Japan; ^4^ Austrian Institute for Health Technology Assessment Vienna Austria; ^5^ Institute for Cancer Control, National Cancer Center Japan Tokyo Japan; ^6^ Department of Mental Health, Institute of Industrial Ecological Sciences University of Occupational and Environmental Health, Japan Kitakyushu Japan; ^7^ Institutional Research Center University of Occupational and Environmental Health, Japan Kitakyushu Japan; ^8^ School of Health Innovation Kanagawa University of Human Services Kawasaki Japan; ^9^ Healthcare Business Division Fujitsu Japan limited Kanagawa Japan; ^10^ Faculty of Human Sciences University of Tsukuba Tokyo Japan; ^11^ Department of Community Mental Health and Law National Institute of Mental Health, National Center of Neurology and Psychiatry Tokyo Japan; ^12^ Department of Clinical Psychology, Faculty of Social Policy & Administration, Hosei University Tokyo Japan; ^13^ Faculty of Policy Management Keio University Japan

**Keywords:** biopsychosocial medicine, endocrine, gynecology, occupational health, reproductive health

## Abstract

**Objectives:**

This systematic review aimed to assess the association between psychosocial factors in the workplace and menstrual abnormalities or fertility, focusing on literature implementing a prospective cohort design.

**Methods:**

We searched MEDLINE, EMBASE, PsycINFO, PsycARTICLES, and Japan Medical Abstracts Society electronic databases for studies published from inception to February 26, 2020, and updated the search in PubMed on May 29, 2024. Inclusion criteria were (P) adult female workers (over 18 years old), (E) presence of adverse psychosocial factors at work, (C) absence of adverse psychosocial factors at work, and (O) any menstrual cycle disorders, menstrual‐related symptoms, or fertility issues. Prospective cohort studies were included. The included studies were summarized descriptively.

**Results:**

Database searching yielded 14,238 abstracts, with nine studies meeting the inclusion criteria. Outcomes included fertility (*n* = 5), irregular menstrual cycle (*n* = 1), early menopause (*n* = 1), endometriosis (*n* = 1), and serum hormones (*n* = 1). Study findings included that women with high job demands and low job control were less likely to conceive, and working over 40 h per week and frequent heavy lifting, and rotating night shift work increased the risk of earlier menopause. Studies on night shift/rotating work and fertility outcomes showed no significant differences.

**Conclusion:**

This review underscores the insufficient high‐level evidence regarding the association of psychosocial factors at work with fertility and menstrual disorders, emphasizing the necessity for future well‐designed studies.

## 1 INTRODUCTION

Menstrual abnormalities are a crucial issue in women's health. Menstrual cycle irregularities increased the risk of poor health outcomes (Cirillo et al., 2016; Huang et al., 2023; Kiconco et al., 2022; Olsson & Olsson, 2020; Y.‐X. Wang et al., 2020; Y.‐X. Wang et al., 2022) and are sometimes caused by sex hormone disturbances, which also affect the fertility outcomes. Many women who hope to be pregnant in the future are workers.

Psychosocial factors in the workplace may disrupt the menstrual cycle and ovarian functions. The International Labour Organization (ILO) defines psychosocial factors in the workplace as those referring to work environment interactions, job content, organizational factors and skills, and individual features of labor that may negatively influence individuals' health and satisfaction with their jobs (ILO, 1986). Some studies showed significant associations: job stress perceptions and low supervisor support with high severity of menstrual symptoms (Bariola et al., 2017; Şen et al., 2023), strenuous work and work schedule with menstrual cycle irregularity (S. Song et al., 2022; Y. Wang et al., 2016). Still, studies that examined the associations of psychosocial factors at work with menstrual disorders showed inconsistent results (Albert‐Sabater et al., 2016; Lim et al., 2016; Minguez‐Alarcon et al., 2017). In terms of infertility, recent cross‐sectional studies reported that night shift workers were more likely to be infertile than day workers (Fernandez et al., 2020; Minguez‐Alarcon et al., 2017). However, another study reported no significant association between job strain and women's fertility status (Sheiner et al., 2003). Psychological chronic stress can impact hormones through an interactive activate/deactivate process between the hypothalamic–pituitary–adrenal (HPA) axis and sex hormones (i.e., the hypothalamic–pituitary‐ovarian [HPO] axis) (Ramya et al., 2023; Roberts et al., 2020). Thus, work‐related factors may potentially cause infertility (Ramya et al., 2023); however, there are limitations to estimating the magnitude of the associations due to the cross‐sectional nature of the studies (Stocker et al., 2014).

As described above, psychosocial factors at work were not thoroughly examined. Also, as most studies adopted a cross‐sectional design (i.e., one cycle was treated as one individual outcome), the possibility of reverse causation cannot be definitively ruled out. For example, high severity of menstrual symptoms may make women sensitive to job stress (Şen et al., 2023), and menopausal symptoms may make women hesitate to mention their conditions to their supervisors (especially male bosses), leading to low social support in the workplace (Bariola et al., 2017). Menstrual cycle irregularity may increase perceptions about strenuous work (S. Song et al., 2022). The association between psychosocial factors at work and menstrual abnormalities or fertility has not yet been systematically presented. Longitudinal studies should be summarized for concrete conclusions.

The aim of this systematic review was thus to investigate whether psychosocial factors at work impact menstrual characteristics or fertility, focusing specially on studies that has utilized a prospective cohort design.

## 2 METHODS

### 2.1 Study design

The method was reported according to the Preferred Reporting Items for Systematic Review and Meta‐Analysis (PRISMA 2020) guidelines (Page et al., 2021). The study protocol has been registered at the UMIN registry (registration number: UMIN000039488 URL: https://upload.umin.ac.jp/cgi-bin/ctr/ctr_view_reg.cgi?recptno=R000044704). The registration date is February 14, 2020. The protocol paper is available elsewhere (Sasaki et al., 2022).

### 2.2 PECO and eligibility criteria

The eligible participants, exposures, comparisons, and outcomes (PECO) of this systematic review are listed in Table 1. PECO was P: adult female workers (over 18 years old), E: adverse psychosocial factors at work (+), C: adverse psychosocial factors at work (−), and O: menstrual disorders, related symptoms, reproductive outcomes. The study design of included studies was limited to a longitudinal or prospective approach only. The study was also excluded if the cross‐sectional analysis method was applied, even if the study used a longitudinal design. The language was limited to English or Japanese (Sasaki et al., 2022).

**TABLE 1:** Eligibility criteria in the present systematic review.

	Inclusion criteria	Exclusion criteria
Design	Prospective cohort design	Outcome was not assessed at least at two time points Menstrual cycle was cross‐sectionally analyzed as an independent unit
Participants	Adult female workers (over 18 years old)	Pregnant workers Clinical students
Exposures	Presence of adverse psychosocial factors at work	N/A
Comparisons	Absence of adverse psychosocial factors at work	N/A
Outcomes	Menstrual disorders and related symptoms: (1) diverse menstrual dysfunctions (e.g., cycle disorder, hypermenorrhea), (2) gynecological disease/syndrome (e.g., endometriosis, polycystic ovarian syndrome), (3) menstrual‐related symptoms (e.g., PMS, menopausal symptoms) Reproductive outcomes (e.g., infertility, time to be pregnant) Biological outcomes (e.g., serum sex hormone)	Pregnancy‐related outcomes (e.g., premature birth) Malignant outcomes (e.g., cancer)
Article type	Original article published by peer‐reviewed journal (including advanced online publication)	Conference abstract Letter/correspondence
Language	Written in English or Japanese	N/A

Abbreviations: NA, not applicable; PMS, premenstrual syndrome.

### 2.3 Search and information sources

Databases including PubMed (MEDLINE), Embase, PsycINFO, PsycARTICLES, and the Japan Medical Abstract Society database were searched for publications from inception to February 26, 2020. The search was also updated in PubMed, limiting the literature from 2020 to May 29, 2024. The details of the search terms are shown in Appendix A. The search terms for psychosocial factors at work were defined based on previous meta‐analyses (Eguchi et al., 2018; Imamura et al., 2019; Sakuraya et al., 2017; Watanabe et al., 2018). The search terms for outcome variables were initially selected by an investigator (NS) who reviewed previous systematic reviews handling menstrual‐related health (Cerqueira et al., 2017; Chiaffarino et al., 2016; Soliman et al., 2016; J. A. Song et al., 2018; Stocker et al., 2014). The medical doctor in obstetrics and gynecology (outside the research members) confirmed the search terms before searching. The terms were amended to exclude infectious, inflammatory disease, and pelvic organ prolapse due to the lack of probability that psychosocial work‐related factors cause these.

### 2.4 Study selection

Microsoft Excel (Washington, USA) was used to manage all identified studies. An investigator (NS) excluded duplicate records. The remaining articles were shared with 16 investigators (NS, KI, KW, EA, HE, AI, KT, YH, YKom, MIi, YO, ASa, YA, MIw, YKob, and RI). A pair of investigators independently assessed the title and abstract of each article according to the eligibility criteria (shifting phase: first screening). Subsequently, two investigators independently reviewed the full texts that were included in the first screening. Any disagreements were discussed until consensus was reached by all authors. The reasons why studies were excluded were recorded during the full‐text review phase.

### 2.5 Data collection process and data items

Data were extracted independently from eligible articles by 16 investigators (NS, KI, KW, EA, HE, AI, KT, YH, YKom, MIi, YO, ASa, YA, MIw, YKob, and RI). An investigator (NS) integrated and formatted the information on publication year, study design, the country of study origin, the number of participants completing the baseline survey and included in the statistical analysis, demographic characteristics of the participants (i.e., age, occupation), the length of follow‐up and attrition rate, exposure variables (i.e., adverse psychosocial factors at work), and outcome variables (i.e., menstrual abnormalities, fertility).

### 2.6 Risk of bias in individual studies

The risk of bias in observational studies of exposures (ROBINS‐I) tool was used to assess the quality of included studies. Investigators (NS, KI, KW, ASa, and YH) independently scored the bias classified as low, high, or unclear. Discrepancies in quality assessment among the investigators were solved by discussion and consensus among all authors.

### 2.7 Synthesis of results and meta‐analysis

For the main analysis, we synthesized all types of psychosocial factors at work and all types of menstrual‐related disorders/symptoms. Meta‐analysis was conducted if at least three eligible studies were found to have the same outcome. If a meta‐analysis was not appropriate (i.e., only two or fewer studies were eligible and included), the results were presented in a narrative format.

### 2.8 Changes to the protocol

To examine the association between exposure and outcome longitudinally, the study excluded any cross‐sectional fashioned analysis (i.e., one menstrual cycle was treated as an individual unit of outcome).

## 3 RESULTS

### 3.1 Database searching

**FIGURE 1:**
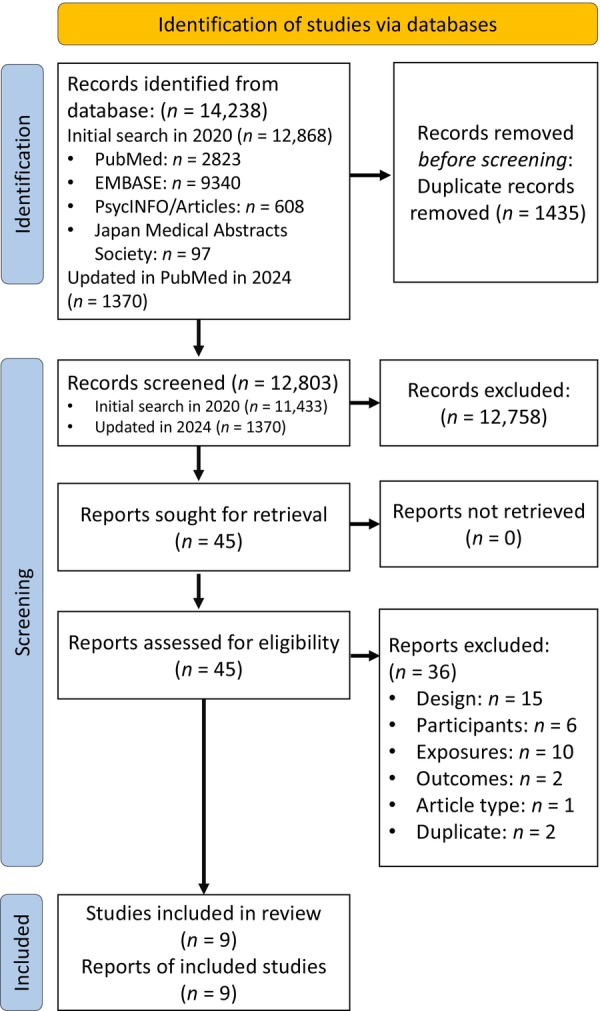
Preferred Reporting Items for Systematic Review and Meta‐Analysis (PRISMA) 2020 flow diagram of systematic review search results.

Database searching yielded 12,868 abstracts (PubMed *n* = 2823, EMBASE *n* = 9340, PsycINFO *n* = 608, Japan Medical Abstracts Society *n* = 97). After removing 1435 duplicates, 11,433 records were included in the first screening, after which 11,395 records were excluded, and 38 records proceeded to full‐text screening. Subsequently, 32 studies that did not meet the criteria for design (*n* = 13), participants (*n* = 4), exposures (*n* = 10), outcomes (*n* = 2), article type (*n* = 1), and duplicates (*n* = 2) were excluded. The list of literature excluded in the second screening is available in Appendix B. Following the updated process in 2024, 1370 records were included in the screening, and 7 records proceeded to full‐text screening. Subsequently, four studies that did not meet the criteria for design (*n* = 2) and participants (*n* = 2) were excluded. Finally, nine studies were included in the systematic qualitative review (Barzilai‐Pesach et al., 2006; Freeman et al., 2023; Gaskins et al., 2015; Kim et al., 2024; Michels et al., 2020; Sabbath et al., 2024; Schernhammer et al., 2011; Stock et al., 2019; Willis et al., 2019). The meta‐analysis was not conducted due to insufficient studies based on our protocol and heterogeneity of outcome measurements. The flowchart of study selection is shown in Figure 1.

### 3.2 Study description

The characteristics of the nine included studies are shown in Table 2. Five studies treated fertility outcomes (Barzilai‐Pesach et al., 2006; Freeman et al., 2023; Gaskins et al., 2015;Sabbath et al., 2024; Willis et al., 2019). Irregular menstrual cycle (Kim et al., 2024), earlier menopause (Stock et al., 2019), incident diagnosis of endometriosis (Schernhammer et al., 2011), and serum reproductive hormone (Michels et al., 2020) were each measured by one study. Three of the nine studies retrieved their data from the Nurse's Health Study (NHS). Eight studies measured shift work as an adverse psychosocial factor at work. Each study reported job demands (Barzilai‐Pesach et al., 2006), job control (Kim et al., 2024), and long working hours (Gaskins et al., 2015; Kim et al., 2024).

**TABLE 2:** The descriptions of the included studies (*N* = 9).

First author, year (country)	Design (cohort name, duration)	Baseline *N* (*N* for analysis)	Participants	Exposure	Comparison	Outcome	F/U (%)	Main results
NHS cohort								
Stock et al. (2019) (United States)	Prospective cohort (NHS2 1989–2011)	116,429 (80,840)	Female registered nurses between ages 25 and 42, who did not experience natural or medically induced menopause; did not use menopausal hormone therapy (HT); or did not have cancer diagnosis (excluding nonmelanoma skin cancer)	Rotating night shift (recent number of months worked rotating night shifts, which defined as working sometime between midnight and 2 a.m.)	Never worked on rotating night shifts	Age at natural menopause over 22 years of follow‐up/earlier menopause younger than 45 years	Every 2 years follow‐up questionnaires were sent to cohort members. Response rates to the NHS 2 questionnaires have been at 90% throughout the follow‐up period	Women who had worked 10–19 months of rotating night shifts had the largest point estimate (HR = 1.11 [95% CI: 1.01–1.21]) and women who had worked ≥20 months had similar result (HR = 1.09 [95% CI: 1.02–1.16]). Cumulative rotating night work of 10 or more years was also associated with a higher risk of menopause among women reaching menopause under age 45 (HR, 10–19 years = 1.22 [95% CI: 1.03–1.44]; HR ≥ 20 years = 1.73 [95% CI: 0.90–3.35]).
Gaskins et al. (2015) (United States and Canada)	Prospective cohort (NHS3 2014–2017)	38,016 (1739)	Female registered nurses trying to get pregnant	Typical work schedule over past year (evenings only/nights only/rotating with nights/rotating no nights); weekly hours of nursing work over past year (1–20 h/>40 h); frequency of night work in past month (≤1/2–3/>3); duration of rotating night shifts (<1 year/1–2 years/>3 years); duration of permanent night shifts (<1 year/1–2 years/>3 years); duration of night shifts (<1 year/1–1.9 years/2–4 years/>4 years); duration of standing/walking(0 or less than 1 h/1–4 h/>8 h); and frequency of moving or lifting a heavy load (1–5/6–15/>15 times)	Typical work schedule over past year (days only); weekly hours of nursing work over past year (21–40 h); frequency of night work in past month (none); duration of rotating night shifts (never); duration of permanent night shifts (never); duration of night shifts (never); duration of standing/walking(5–8 h); frequency of moving or lifting a heavy load (none)	The current duration of their ongoing pregnancy attempt.	Every 6 months (no data available)	Working over 40 h per week was related to increasing 20% (95% CI: 7%–35%) the median duration of pregnancy attempt compared with working 21–40 h/week (*p* = .006). After adjustment for demographic and work‐related confounders, women lifting or moving a heavy load >15 times per day had a 49% (95% CI: 20%–85%) longer median duration of pregnancy attempt (*p* = .002). Typical work schedules over the past year, frequency of night work in the past month and duration of rotating, or nonrotating night shifts were not also significantly associated with fecundity.
Schernhammer et al. (2011) (United States)	Prospective cohort (NHS 2, 1989–2005)	89,400 (2062)	Female registered nurses of ages 25–42 years, without the diagnosis of endometriosis or a history of infertility at baseline.	Duration of work on rotating night shift (1–4.9 years/≧5 years)	Never worked on rotating night shifts	Diagnosis of endometriosis by laparoscopic confirmation	Every 2 years, follow‐up questionnaires were sent to cohort members. Response rates to the NHS II questionnaires have been at 90% throughout the follow‐up period.	Overall, there was no association between rotating night shift work and risk of endometriosis. When the cases were categorized by infertility status, risk was elevated among women with concurrent infertility and ≥5 years of rotating night shift work (RR = 1.71 [95% CI: 1.18–2.49]; *p*_trend_ = .005), compared with women with no rotating night shift work.
PRESTO cohort								
Willis et al. (2019) (Canada and United States)	Prospective cohort (PRESTO)	8772 (6873)	Women aged 21–45 years, attempting pregnancy, who are not using contraception or fertility treatment	Working rotating shifts (hours that shift in a predictable way from day‐to‐day, week‐to‐week, or month‐to‐month) in the past month	No night/shift work	Fecundability (per‐cycle probability of conception)	Every 8 weeks or until reported conception, initiation of fertility treatment, cessation of pregnancy attempts (86.2%^a^)	No association between shift work and fecundability
Sabbath et al. (2024) (Canada and United States)	Prospective cohort (PRESTO: 2018–2022)	7114 (3110)	Working people who were self‐identified as female, aged 21–45 years, reported having a male partner and reported 6 or fewer cycles of pregnancy attempts without the use of fertility treatment at the enrollment	Job control (low), measured by O*NET (a job exposure matrix). The items of job independence and freedom to make decisions from O*NET were used to assess individuals' job control	Job control (high)	Time to pregnancy (fecundability)	Every 8 weeks for up to 12 months or until reported pregnancy, whichever occurred first (89%)	Only the second lowest quartile of lower job independence was associated with reduced fecundability, with a fecundability ratio (FR) of 0.84 [95% CI: 0.74–0.95]. No significant association between job control (lower job freedom) was seen.
Other cohort								
Michels et al. (2020) (United States)	Prospective cohort (The BioCycle Study, 2005–2007).	259 (259)	Healthy, regularly menstruating women aged 18–44 years, who did not used oral contraceptives, other medications or vitamins; had not been pregnant in the last six months; did not have any diagnoses of chronic conditions; and did not have gynecologic surgeries	With night/shift work	Without night/shift work	Reproductive hormone	Eight times across one to two menstrual cycle (100%)	Women reporting night/shift work (*n* = 77) had lower testosterone (percent difference = −9.9% [95% CI: −18.4 to −0.4]). No significant relationship in estradiol, ovulatory LH, FSH, ovulatory FSH, luteal progesterone, and the risk of sporadic anovulation.
Barzilai‐Pesach et al. (2006) (Israel)	Prospective cohort (1999–2000)	160 (75)	Female working patients with a fertility problem, who attended the fertility and in‐vitro fertilization clinics at the Soroka University Medical Center	Job demands (high), type of job (full‐time job), decision latitude (minimal), shift work (yes)	Job demands (low), Type of job (part‐time), decision latitude (high), shift work (no)	Conception in which the data was obtained from the clinical records	18 months (100%)	Women with high job demand were less likely to conceive (RR = 0.60 [95% CI: 0.42–0.96]). No significant association of decision latitude, nor shift work with conception
Kim et al. (2024) (South Korea)	Longitudinal study (2012–2019)	87,147 (41,516)	Women aged 18 or more who had a job and underwent comprehensive health examinations at Kangbuk Samsung Hospital healthcare centers and had no irregular menstrual cycle at the baseline	With night shift work	Without night shift work	Development of irregular menstrual cycle	Until the development of irregular menstrual cycle or till the end of 2019 (a maximum of 8 years). (61.4%^b^)	The risk of irregular menstruation did not increase in the time span of 0–3 years but increased statistically significantly in the time span of 3–6 years with adjusted HRs (AHRs) of 1.54 (95% CI: 1.40–1.69). In the time span of more than 6 years, the AHRs further increased to 1.95 (95% CI: 1.61–2.35)
Freeman et al. (2023) (United States)	Prospective cohort Secondary analysis of an RCT (EAGeR)	1184 (1177)	Women aged 18–40 with a history of 1–2 pregnancy losses who were actively attempting to conceive again and had a job	With night shift work With rotating shiftwork	Without night shift work Without rotating shiftwork	Time to pregnancy (fecundability)	Up to a maximum of 6 menstrual cycles or pregnancy. The follow‐up rate was more than 90%	Night shift work and rotating shiftwork were not associated with pregnancy (vs. nonnight shift work/rotating shiftwork fecundability) Odds ratios were 1.17 (95% CI: 0.96–1.42) and 1.08 (95% CI: 0.88 1.34), respectively

Abbreviations: CI, confidence interval; EAGeR, Effects of Aspirin in Gestation and Reproduction study; F/U, Follow‐up; HR, hazard ratio; NHS, The Nurses' Health Study; PRESTO, Pregnancy Study Online; RR, relative risk.

^a^ The authors calculated 5926/6873 × 100.

^b^ The authors calculated 41,516/(87,147–19,514) × 100.

Although the authors designed the protocol to conduct a meta‐analysis if we found at least three eligible studies with the same outcome, but fertility outcomes of the included studies varied and it was difficult to synthesize the findings: time to pregnancy (Sabbath et al., 2024; Willis et al., 2019), the current duration of participants' ongoing pregnancy attempt (Gaskins et al., 2015), time to positive urine hCG pregnancy tests (Freeman et al., 2023), and conception (Barzilai‐Pesach et al., 2006).

Regarding fertility outcomes, Barzilai‐Pesach et al. (2006) prospectively studied the association between women's occupational stress factors (job demands, decisional latitude, shift work, and working hours) and fertility treatment outcomes in 75 women (Barzilai‐Pesach et al., 2006). High job demands reduced the chances of conception (relative risk [RR] = 0.60, [95% confidence interval [CI]: 0.42–0.96]). Gaskins et al. (2015) examined the impact of work schedule and physical factors on fecundity in 1739 female nurses from the NHS 3 cohort. Longer working hours (>40/week) and heavy lifting (>15 times/day) extended median pregnancy attempt duration by 20% [95% CI: 7%–35%] and 49% [95% CI: 20%–85%], respectively. This effect was more pronounced in overweight or obese women. Night shift work over the past year was not significantly associated with fecundity. Willis et al. (2019) prospectively assessed shift work's impact on fecundability in 6873 North American women. No association between shift work and fecundability was found. Freeman et al. (2023) examined the impact of night shift work on fecundability among 1177 working women with a history of one to two pregnancy losses who were attempting to conceive again. No association between night shift work and fecundability was found. Sabbath et al. (2024) prospectively assessed job control (operationalized as job independence and freedom to make decisions) and time to pregnancy among 3110 female workers and reported ≤6 cycles of pregnancy attempt time at enrollment in the Pregnancy Study Online (PRESTO) cohort (2018–2022). A J‐shaped relationship between job independence and fecundability was observed; second from lowest quartiles showed shortest time to pregnancy (fecundability ratios = 0.84 [95% CI: 0.74–0.95], as reference of highest quartiles). No significant association was found in the association between freedom to make decisions and fecundability.

Stock et al. (2019) prospectively evaluated the association between nurses' rotating night shift work (i.e., the number of months with at least three nights per month in the past 2 years) and menopausal age, using the data from an ongoing prospective cohort study (NHS 2) (Stock et al., 2019). This cohort included 116,429 female registered nurses in the United States, aged between 25 and 42 years, at the baseline survey in 1989, followed from 1991 through 2013. Participants were asked whether their menstrual periods had ceased, at what age, and if this was due to natural menopause, chemotherapy or radiation, or surgery. Of those whose menstrual periods had not stopped before the last follow‐up, 27,456 had natural menopause. The mean age of natural menopause in these women was 50 (SD ±4.0) years. Among them, 2524 had menopause under 45 years. Significant associations were found between the number of months of night shift work in the past 2 years and earlier menopause (HRs = 1.06–1.11 among women with <10 months, 10–19 months, and with ≥20 months, compared with none of night shift work). Cumulative rotating night work of 10 or more years was also associated with a higher risk of menopause among women reaching menopause under age 45. These analyses did not consider any confounders such as psychological stress, job strain, or chronotype‐related tolerance.

Schernhammer et al. (2011) reported an association between rotating night shift work and endometriosis risk within the NHS 2 (Schernhammer et al., 2011). Participants were asked whether they had ever had physician‐diagnosed endometriosis. Analyses of incident diagnosis of endometriosis were restricted to those women who reported laparoscopic confirmation of their diagnosis. This study included 2062 laparoscopically confirmed cases documented during 16 years of follow‐up evaluation from 89,400 women without diagnosed endometriosis at baseline. Overall, there was no association between rotating night shift work and endometriosis. When the cases were categorized by infertility status, the risk was elevated among women with concurrent infertility and ≥5 years of rotating night shift work (RR = 1.71 [95% CI: 1.18–2.49]; *p*_trend_ = .005), compared with women with no rotating night shift work.

Michels et al. (2020) examined the influences of night/shift work on serum reproductive hormones. The participants were recruited in the United States from the BioCycle Study (2005–2007), a prospective cohort of 259 healthy women (mean age = 24) not using oral contraceptives. At baseline, participants were asked whether they were currently employed and whether they worked nights or rotating shifts. Fasting blood samples were collected at eight clinic visits across each menstrual cycle. Fertility monitors were used to assist in the timing of mid‐cycle clinic visits. Two cycles were followed. The proportion of participants who had night/shift work was 34.4%. The results showed that night/shift work did not influence estradiol or progesterone. Night/shift workers showed lower testosterone (−9.9%) than employees without night/shift work, but the clinical implication of the finding was not mentioned.

Kim et al. (2024) conducted an 8‐year follow‐up cohort study to investigate the association between night shift work and irregular menstrual cycles among female workers. The study included 41,516 women in the longitudinal analysis from the Kangbuk Samsung Health Study (2012–2019). At baseline, participants were asked about night shift work. The longitudinal analysis found an increased risk of developing irregular menstrual cycles among night shift workers (adjusted HR = 1.95 [95% CI: 1.61–2.35]) after 6 years. No significant differences were observed among subgroups stratified by sleep quality, working hours, or obesity; the longitudinal association between night shift work and the development of irregular menstrual cycle was consistent regardless of these three factors.

### 3.3 Risk of bias assessment

The summary of the risk of bias assessment by ROBINS‐I is shown in Table 3. The overall judgment was all “serious” due to confounding or selection of reported results.

**TABLE 3:** Summary of risk of bias assessment for the included studies analyzed by ROBINS‐I (*N* = 9).

	Stock et al. (2019)	Gaskins et al. (2015)	Schernhammer et al. (2011)	Willis et al. (2019)	Sabbath et al., 2024	Michels et al. (2020)	Barzilai‐Pesach et al. (2006)	Kim et al. (2024)	Freeman et al. (2023)
Confounding	M	S	S	S	S	S	S	M	S
2 Selection of participants	L	L	S	L	L	L	S	L	L
3 Classification of intervention	M	M	S	M	L	M	S	M	M
4 Deviations from intended interventions	L	L	L	L	L	L	L	L	L
5 Missing data	S	L	S	M	M	S	S	S	L
6 Measurement of outcomes	S	S	L	S	S	L	L	S	L
7 Selection of reported result	S	S	S	M	M	S	S	S	S
Overall judgment	S	S	S	S	S	S	S	S	S

*Note*: Different colour shades reflect the risk of bias.

Abbreviations: C, critical risk of bias; L, low risk of bias; M, moderate risk of bias; N, no information; ROBINS‐I, The risk of bias in observational studies of exposures; S, serious risk of bias.

## 4 DISCUSSION

All four studies (Barzilai‐Pesach et al., 2006; Freeman et al., 2023; Gaskins et al., 2015; Willis et al., 2019) that investigated the risk of shift/night work for pregnancy outcomes showed nonsignificant associations. These studies were not included in the recent meta‐analysis published in 2014 (Stocker et al., 2014) that examined the influence of shift work on reproductive outcomes, showing that shift work increased the risk of infertility (crude odds ratio [OR]: 1.80 [95% CI: 1.01–3.20]). This meta‐analysis included four cross‐sectional fashioned studies (one study was a retrospective cohort) from 1996 to 2003 (Bisanti et al., 1996;Spinelli et al., 1997; Tuntiseranee et al., 1998; Zhu et al., 2003). These studies retrieved a pregnant population and obtained the occupational data retrospectively. Three of them did not show significant associations (Spinelli et al., 1997; Tuntiseranee et al., 1998; Zhu et al., 2003) and one did (Bisanti et al., 1996). Certainly, some studies seemed to find that women with night/shift work tend to take a long time to become pregnant (Spinelli et al., 1997; Tuntiseranee et al., 1998; Zhu et al., 2003). However, the significance disappeared after adjusting the demographic and job characteristics information. The adjusted results of the meta‐analysis also showed nonsignificant associations (adjusted OR: 1.12 [0.86–1.44]) (Stocker et al., 2014). From both our findings and previous reviews, we cannot conclude that shift/night work itself has an influence on fertility. More studies should be conducted using rigorous longitudinal study designs that account for possible confounding factors (psychosocial factors at work or lifestyle factors) to examine the association between shift work and fertility outcomes.

Three studies examined psychosocial factors at work other than shift/night work. These studies showed that low job control, high job demands, long working hours, and heavy lifting were significantly associated with infertility (Barzilai‐Pesach et al., 2006; Gaskins et al., 2015; Sabbath et al., 2024). These findings were consistent with previous cross‐sectional studies that were not included in the present review, which suggested that long working hours and physically demanding jobs lead to less ovarian function and infertility (Minguez‐Alarcon et al., 2017; Tuntiseranee et al., 1998). In contrast, another study reported a nonsignificant association (Spinelli et al., 1997). Longitudinal studies included in this study suggested the importance of focusing on psychosocial factors at work for women's health.

As a potential mechanism, psychological stress reactions (i.e., depression, low positive affect) declined ovarian function (Bleil et al., 2012); whether these psychosocial factors at work affect fertility through such distress or not (direct impact) needs to be examined. Psychological stress theoretically disrupts HPA–HPO axis interactions (Lennartsson et al., 2012) and has been associated with higher levels of oxidative stress, which potentially declines ovarian function. Regarding physical demands, jobs with high intensity (energy expenditure per working day) and fatigue (energy expenditure per working hour) reduced fecundity (Florack et al., 1994). A previous study suggested that during times of high workloads in our evolutionary past, limited energy intake caused a negative energy balance, leading to 'pre‐emptive ovarian suppression.' This response persists even when energy intake is later compensated (Jasieńska & Ellison, 1998). However, robust studies investigating the associations between psychosocial factors at work and fertility outcomes are lacking. Further prospective studies are needed in this area.

One study in this review showed an increased risk of early menopause from shift/night work (Stock et al., 2019). Rotating night shift work experience in the prior 2 years increased the risk of earlier menopause. The subgroup analysis with women under 45 showed that over 10 years of cumulative rotating night shift work exposure increased the risk of early menopause (Stock et al., 2019). This finding is consistent with the recent systematic review that included cross‐sectional studies investigating job stress and menopause (Martelli et al., 2021); the authors reported that age at menopause and severity of menopausal symptoms were both influenced by job‐related factors such as high job strain. The review identified the risk factor for early menopause as smoking, type of work (e.g., service work), and shift work. The mechanism by which night shift work leads to early menopause is explained by several potential biological factors, such as an adverse impact of circadian disruption on ovulation, melatonin as an antiapoptotic agent within the follicle, and higher levels of oxidative stress induced by psychological stress (Stock et al., 2019). Early menopause has been regarded as a significant outcome because it increases the risk of cardiovascular disease and all‐cause mortality (Muka et al., 2016). Further investigations are needed to prevent the deterioration of female workers' health if any occupational factors cumulatively affect early menopause.

In this review, one study showed no significant association of rotating night shift work with the onset of endometriosis diagnosed by laparoscopy (Schernhammer et al., 2011). Night shift work was found to decrease melatonin levels (Burch et al., 2005), reduced aromatase activity, and worked as a potential antiestrogenic agent (Cos et al., 2005). Elevated estrogen levels caused by low melatonin levels from night shift work could promote endometriosis growth. A population‐based case–control study reported that any night shift work was associated with a 48% increased risk of endometriosis (Marino et al., 2008). The authors of the included study discussed the possible bias that women with clinical pain symptoms or with children might be likely to be out of the workforce in rotating work. Additionally, laparoscopic confirmation of endometriosis among those who never experienced infertility is restricted to those with clinical symptoms, leading to underestimating the risk in those who never experienced infertility. The associations of shift work with endometriosis need further investigation.

One study from South Korea showed an increased risk of irregular menstrual cycles from night shift work after 6 years (Kim et al., 2024). A potential mechanism was discussed in the paper that hormonal changes due to circadian disruption caused by exposure to light at night were likely to be occurred by cumulative exposure to night shift work. Interestingly, the positive association of shift work with menstrual abnormality has been often reported in cross‐sectional studies from Asia (Kwak & Kim, 2018; Mayama et al., 2020; Ok et al., 2019; S. Su et al., 2008; Y. Wang et al., 2016) but not in Europe (Moen et al., 2015). Geographical sunlight exposure affects circadian rhythms, genetic differences influence irregular menstrual cycles, and sociocultural factors impact occupational characteristics like workplace culture, social mitigation, and night shift conditions (Kim et al., 2024). However, few studies have investigated the association of psychosocial factors at work with menstrual abnormality using longitudinal data. Further study is needed.

This review was limited to studies that evaluated the outcome (menstrual abnormality) at both baseline and follow‐up. Furthermore, studies that analyzed the menstrual cycle as one unit of outcome were excluded. Few studies that met these criteria were found in this review. Some of the studies analyzed the cycle data as one unit (Albert‐Sabater et al., 2016; Fenster et al., 1999; Hjollund et al., 1998; S. B. Su et al., 2008). This approach did not allow for a before/after comparison of data from one person and was considered a cross‐sectional study. Menstrual abnormality can be caused not only by occupational stress but also by multiple other factors (e.g., body mass index, nonoccupational stress). A longitudinal study evaluating the change in menstrual abnormalities is needed to estimate the risk of psychosocial factors at work. Considering the current ongoing academic achievement in this area and its potentially short‐term responsive mechanism (e.g., HPA–HPO interactions), cross‐sectional studies still have their usefulness. However, a strength of the longitudinal design is that it can avoid reverse causation. Also, the exact time between exposure and the onset of outcomes has not yet been confirmed in this area. Collecting data longitudinally may deepen the understanding of this association.

### 4.1 Strengths and limitations

This systematic review was the first review to investigate the evidence of the associations between psychosocial factors at work and menstrual abnormalities or fertility, focusing specially on the longitudinal well‐designed studies. However, this systematic review has several limitations. First, we did not conduct a meta‐analysis due to an insufficient number of studies. Thus, concrete evidence was not provided for the clinical questions. Second, this review is restricted to English and Japanese languages; therefore, studies in other languages may have been missed. Third, publication bias can result from language limitation and exclusion of presentation abstracts, but this review did not assess that bias. Fourth, risk of bias was assessed by using ROBINS‐I in this study following our protocol (Sasaki et al., 2022) because the risk of bias in non‐randomized studies ‐ of exposure (ROBINS‐E) was not available at the start of this review. Although ROBINS‐I is applicable to all nonrandomized trials and aligns with the Grading of Recommendations Assessment, Development and Evaluation system, the use of ROBINS‐E should be considered in the future. In addition, we acknowledge that the included studies were of low quality. Fifth, observational studies are less able to provide evidence of causality than intervention studies.

## 5 CONCLUSIONS AND IMPLICATIONS

This systematic review investigated the association of psychosocial factors at work with menstrual abnormalities and fertility outcomes, and nine articles were included. This review presented insufficient high‐level evidence of well‐designed studies on this topic. Because psychosocial factors at work are modifiable risk factors for women's reproductive outcomes, further studies are needed to determine the implications for occupational health practice. This study highlights the importance of considering psychosocial factors at work to improve the health of working women.

## AUTHOR CONTRIBUTIONS

Natsu Sasaki, Kotaro Imamura, Kazuhiro Watanabe, Emiko Ando, Hisashi Eguchi, Akiomi Inoue, Kanami Tsuno, Yui Hidaka, Yu Komase, Mako Iida, Yasumasa Ootsuka, Asuka Sakuraya, Mai Iwanaga, Yuka Kobayashi, and Reiko Inoue contributed to shifting, full‐text review, and extraction of information from each of the included studies for systematic review. Natsu Sasaki, Kotaro Imamura, Kazuhiro Watanabe, Asuka Sakuraya, and Yui Hidaka independently assessed the included study quality using the risk of bias assessment tool. All authors conceived the study, developed the study design, prepared the first draft, and approved the final manuscript.

## CONFLICT OF INTEREST STATEMENT

Natsu Sasaki reports personal fees from Medilio Inc., outside the submitted work. Kotaro Imamura, Asuka Sakuraya, and Norito Kawakami are employed at the Department of Digital Mental Health, an endowment department supported with an unrestricted grant from 15 enterprises (https://dmh.m.u-tokyo.ac.jp/c), outside the submitted work.

## APPENDIX A

### A.1 Search terms for PubMed

(employe*[tw] OR manag*[tw] OR colleague*[tw] OR worksit*[tw] OR “work”[tw] OR works*[tw] OR work'*[tw] OR worka*[tw] OR worke*[tw] OR workg*[tw] OR worki*[tw] OR workl*[tw] OR workp*[tw] OR occupant*[tw] OR company*[tw] OR offic*[tw] OR busines*[tw] OR workplace[mh]) AND (("Stress, Mechanical"[Mesh] OR "Lifting"[Mesh] OR "Moving and Lifting Patients"[Mesh] OR "Weight‐Bearing"[Mesh] OR "Biomechanics" OR "Physical Exertion"[Mesh] OR "Torsion, Mechanical"[Mesh] OR "Postural Balance"[Mesh] OR "Walking"[Mesh] OR "Recovery of Function"[Mesh] OR "Relaxation"[Mesh] OR (static[Title/Abstract] AND posture) OR (awkward[Title/Abstract] AND posture) OR (dynamic[Title/Abstract] AND posture) OR static work[Title/Abstract] OR dynamic load*[Title/Abstract] OR lift*[Title/Abstract] OR carry*[Title/Abstract] OR hold*[Title/Abstract] OR pull*[Title/Abstract] OR drag*[Title/Abstract] OR push*[Title/Abstract] OR manual handling[Title/Abstract] OR force*[Title/Abstract] OR biomechanic*[Title/Abstract] OR walking*[Title/Abstract] OR postural balance[Title/Abstract] OR flexion*[Title/Abstract] OR extension*[Title/Abstract] OR turning[Title/Abstract] OR sitting[Title/Abstract] OR kneeling[Title/Abstract] OR squatting[Title/Abstract] OR twisting[Title/Abstract] OR bending[Title/Abstract] OR reaching[Title/Abstract] OR standing[Title/Abstract] OR sedentary[Title/Abstract] OR repetitive movement*[Title/Abstract] OR monotonous work[Title/Abstract] OR relaxation[Title/Abstract] OR recovery of function[Title/Abstract] OR physical demand*[Title/Abstract] OR physically demand*[Title/Abstract]) OR ("Stress, Psychological"[Majr] OR "Social Support"[Majr] OR "Job Satisfaction"[Mesh] OR "Work Schedule Tolerance"[Mesh] OR "Employee Performance Appraisal"[Mesh] OR "Employee Grievances"[Mesh] OR "Social Justice/psychology"[Mesh] OR "Personnel Downsizing"[Mesh] OR "Staff Development"[Mesh] OR "Organizational Culture"[Mesh] OR "Bullying"[Mesh] OR "Prejudice"[Mesh] OR "Social Discrimination"[Mesh] OR "Interpersonal Relations"[Mesh] OR "Communication/psychology"[Mesh]) OR (psychosocial[Title/Abstract] OR job strain[Title/Abstract] OR work strain[Title/Abstract] OR work demand*[Title/Abstract] OR job demand*[Title/Abstract] OR high demand*[Title/Abstract] OR low control[Title/Abstract] OR lack of control[Title/Abstract] OR work control[Title/Abstract] OR job control[Title/Abstract] OR decision latitude[Title/Abstract] OR work influence*[Title/Abstract] OR demand resource*[Title/Abstract] OR effort reward*[Title/Abstract] OR time pressure*[Title/Abstract] OR recuperation*[Title/Abstract] OR work overload*[Title/Abstract] OR work over‐load*[Title/Abstract] OR recovery[Title/Abstract] OR coping[Title/Abstract] OR work ability[Title/Abstract] OR social support[Title/Abstract] OR support system*[Title/Abstract] OR social network*[Title/Abstract] OR emotional support[Title/Abstract] OR interpersonal relation*[Title/Abstract] OR interaction*[Title/Abstract] OR social capital [Title/Abstract] OR justice*[Title/Abstract] OR injustice*[Title/Abstract] OR job satisfaction[Title/Abstract] OR work satisfaction[Title/Abstract] OR boredom[Title/Abstract] OR skill discretion*[Title/Abstract] OR staff development[Title/Abstract] OR discrimination[Title/Abstract] OR harass*[Title/Abstract] OR work‐place conflict*[Title/Abstract] OR workplace violen*[Title/Abstract] OR work‐place violen*[Title/Abstract] OR bullying[Title/Abstract] OR ageism[Title/Abstract] OR homophobia[Title/Abstract] OR racism[Title/Abstract] OR sexism[Title/Abstract] OR victimization*[Title/Abstract] OR silent workplace*[Title/Abstract] OR role ambiguity[Title/Abstract] OR role‐conflict*[Title/Abstract] OR work‐role*[Title/Abstract] OR working hour*[Title/Abstract] OR working time[Title/Abstract] OR day‐time[Title/Abstract] OR night‐time[Title/Abstract] OR shift work*[Title/Abstract] OR work shift*[Title/Abstract] OR temporary work[Title/Abstract] OR full‐time[Title/Abstract] OR part‐time[Title/Abstract] OR flexible work*[Title/Abstract] OR organizational change[Title/Abstract] OR organisational change[Title/Abstract] OR lean production[Title/Abstract] OR job security[Title/Abstract] OR job insecurity[Title/Abstract])) AND (menstruation OR menstrual OR “menstrual pain” OR “period pain” OR “period pains” OR menorrhagia OR hypermenorrhea OR dysmenorrhea[MeSH] OR premenstrual OR “PMS” OR “premenstrual syndrome” OR “PMDD” OR “premenstrual dysphoric disorder” OR amenorrhea OR menoxenia OR metrorrhagia OR endometrio* OR myoma* OR leiomyoma OR fibroids OR adenomyosis OR ovarian cyst* OR endometrioma OR polycystic ovar* OR menopaus* OR hot flush* OR climacteric OR estrogen* OR estradiol OR progesterone* OR prolactin OR lutenizing OR LH OR “lutenizing hormone” OR follicul* OR FSH OR “follicle stimulating hormone” OR gonadotropin OR GnRH OR “gonadotropin releasing hormone” OR “basal body temperature” OR infertility OR fertility OR fecund* OR subfecundity OR reproductiv*) AND (longitudinal OR prospective OR cohort OR (follow AND up) OR observational OR (case AND (control or cohort)) OR case‐control OR case‐cohort).

## APPENDIX B THE LIST OF EXCLUDED LITERATURE (*n* = 36)

### B.1 Did not meet the criteria for design (*n* = 15)

Albert‐Sabater, J. A., Martinez, J. M., Baste, V., Moen, B. E., & Ronda‐Perez, E. (2016). Comparison of menstrual disorders in hospital nursing staff according to shift work pattern. *Journal Clinical Nurses, 25*(21‐22), 3291–3299. doi: 10.1111/jocn.13371

Fenster, L., Waller, K., Chen, J., Hubbard, A. E., Windham, G. C., Elkin, E., & Swan, S. (1999). Psychological stress in the workplace and menstrual function. *American Journal of Epidemiology, 149*(2), 127–134. doi: 10.1093/oxfordjournals.aje.a009777

Hjollund, N. H., Kold Jensen, T., Bonde, J. P., Henriksen, T. B., Kolstad, H. A., Andersson, A. M., Ernst, E., Giwercman, A., Skakkebaek, N. E., & Olsen, J. (1998). Job strain and time to pregnancy. *Scandinavian Journal of Work Environmental Health, 24*(5), 344–350. doi: 10.5271/sjweh.354

Hourani, L. L., Yuan, H. X., & Bray, R. M. (2004). Psychosocial and lifestyle correlates of premenstrual symptoms among military women. *Journal of Women's Health, 13*(7), 812–821. doi: 10.1089/jwh.2004.13.812

Kim, O., Ahn, Y., Lee, H. Y., Jang, H. J., Kim, S., Lee, J. E., Jung, H., Cho, E., Lim, J. Y., Kim, M. J., Willett, W. C., Chavarro, J. E., & Park, H. Y. (2017). The Korea Nurses' Health Study: A prospective cohort study. *Journal of Womens Health (Larchmt), 26*(8), 892–899. doi: 10.1089/jwh.2016.6048

Lim, A. J., Huang, Z., Chua, S. E., Kramer, M. S., & Yong, E. L. (2016). Sleep duration, exercise, shift work and polycystic ovarian syndrome‐related outcomes in a healthy population: A cross‐sectional study. *PLoS One, 11*(11), e0167048. doi: 10.1371/journal.pone.0167048

Messing, K., Saurel‐Cubizolles, M. J., Bourgine, M., & Kaminski, M. (1992). Menstrual‐cycle characteristics and work conditions of workers in poultry slaughterhouses and canneries. *Scandinavian Journal of Work Environmental Health, 18*(5), 302–309. doi: 10.5271/sjweh.1572

Minguez‐Alarcon, L., Souter, I., Williams, P. L., Ford, J. B., Hauser, R., Chavarro, J. E., Gaskins, A. J., & Earth Study Team. (2017). Occupational factors and markers of ovarian reserve and response among women at a fertility centre. *Occupational Environmental Medicine, 74*(6), 426–431. doi: 10.1136/oemed‐2016‐103953

Schernhammer, E. S., Rosner, B., Willett, W. C., Laden, F., Colditz, G. A., & Hankinson, S. E. (2004). Epidemiology of urinary melatonin in women and its relation to other hormones and night work. *Cancer Epidemiology Biomarkers & Prevention, 13*(6), 936–943.

Smith, D., Wei, N., & Wang, R. (2005). Subjective health complaints among female hospital nurses in Mainland China. *Journal of Occupational Health Safety (Aust NZ), 21*, 51–59.

Su, S. B., Lu, C. W., Kao, Y. Y., & Guo, H. R. (2008). Effects of 12‐hour rotating shifts on menstrual cycles of photoelectronic workers in Taiwan. *Chronobiology International, 25*(2), 237–248. doi: 10.1080/07420520802106884

Tissot, F., & Messing, K. (1995). Perimenstrual symptoms and working conditions among hospital workers in Quebec. *American Journal of Industrial Medicine, 27*(4), 511–522. doi: 10.1002/ajim.4700270405

Wang, Y., Gu, F., Deng, M., Guo, L., Lu, C., Zhou, C., Chen, S., & Xu, Y. (2016). Rotating shift work and menstrual characteristics in a cohort of Chinese nurses. *BMC women's health, 16*(1), 24. doi: 10.1186/s12905‐016‐0301‐y

Li, L., Lv, X., Li, Y., Zhang, X., Li, M., & Cao, Y. (2023). Development and validation of risk prediction model for premenstrual syndrome in nurses: Results from the nurses‐based the TARGET cohort study. *Frontiers in Public Health*, 11, 1203280.

Ozeki, C., Maeda, E., Hiraike, O., Nomura, K., & Osuga, Y. (2024). Changes in menstrual symptoms and work productivity after checklist‐based education for premenstrual syndrome: An 8‐month follow‐up of a single‐arm study in Japan. *BMC Women's Health*, 24(1), 242.

### B.2 Did not meet the criteria for participants (*n* = 6)

Burdorf, A., Brand, T., Jaddoe, V. W., Hofman, A., Mackenbach, J. P., & Steegers, E. A. (2011). The effects of work‐related maternal risk factors on time to pregnancy, preterm birth and birth weight: The Generation R Study. *Occupational Environmental Medicine, 68*(3), 197–204. doi: 10.1136/oem.2009.046516

Jeyaseelan, L., & Rao, P. S. (1995). Effect of occupation on menstrual cycle length: causal model. Human Biology, 67(2), 283–290.

Lawson, C. C., Johnson, C. Y., Chavarro, J. E., Lividoti Hibert, E. N., Whelan, E. A., Rocheleau, C. M., Grajewski, B., Schernhammer, E. S., Rich‐Edwards, J. W. (2015). Work schedule and physically demanding work in relation to menstrual function: The Nurses' Health Study 3. *Scandinavian Journal of Work Environmental Health, 41*(2), 194–203. doi: 10.5271/sjweh.3482

Wang, X. S., Travis, R. C., Reeves, G., Green, J., Allen, N. E., Key, T. J., Roddam, A. W., & Beral, V. (2012). Characteristics of the Million Women Study participants who have and have not worked at night. *Scandinavian Journal of Work Environmental Health, 38*(6), 590–599. doi: 10.5271/sjweh.3313

McKinnon, C. J., Hatch, E. E., Orta, O. R., Rothman, K. J., Eisenberg, M. L., Wefes‐Potter, J., & Wise, L. A. (2022). The association between work hours, shift work, and job latitude with fecundability: A preconception cohort study. *Journal of Occupational Health Psychology*, *27*(2), 258.

Sponholtz, T. R., Bethea, T. N., Ruiz‐Narváez, E. A., Boynton‐Jarrett, R., Palmer, J. R., Rosenberg, L., & Wise, L. A. (2021). Night shift work and fecundability in late reproductive‐aged African American women. *Journal of Women's Health (Larchmt)*, *30*(1), 137–144. doi: 10.1089/jwh.2019.8166

### B.3 Did not meet the criteria for exposures (*n*=10)

Assadi, S. N. (2013). Is being a health‐care worker a risk factor for women's reproductive system? *International Journal of Preventive Medicine, 4*(7).

Davydov, D. M., Shapiro, D., Goldstein, I. B., & Chicz‐DeMet, A. (2005). Moods in everyday situations: Effects of menstrual cycle, work, and stress hormones. *Journal of Psychosomatic Research, 58*(4), 343–349. doi: 10.1016/j.jpsychores.2004.11.003

Gaskins, A. J., Chavarro, J. E., Rich‐Edwards, J. W., Missmer, S. A., Laden, F., Henn, S. A., & Lawson, C. C. (2017). Occupational use of high‐level disinfectants and fecundity among nurses. *Scandinavian Journal of Work Environmental Health, 43*(2), 171–180. doi: 10.5271/sjweh.3623

Geukes, M., Anema, J. R., van Aalst, M. P., de Menezes, R. X., & Oosterhof, H. (2019). Improvement of menopausal symptoms and the impact on work ability: A retrospective cohort pilot study. *Maturitas, 120*, 23–28. doi: 10.1016/j.maturitas.2018.10.015

Kuczmierczyk, A. R., Labrum, A. H., & Johnson, C. C. (1992). Perception of family and work environments in women with premenstrual syndrome. *Journal of Psychosomatic Research, 36*(8), 787–795. doi: 10.1016/0022‐3999(92)90137‐q

Potter, J., Bouyer, J., Trussell, J., & Moreau, C. (2009). Premenstrual syndrome prevalence and fluctuation over time: Results from a French population‐based survey. *Journal of Women's Health (Larchmt), 18*(1), 31–39. doi: 10.1089/jwh.2008.0932

Sun, S., & Chen, F. (2017). Women's employment trajectories during early adulthood in urban China: A cohort comparison. *Social Science Research, 68*, 43–58. doi: 10.1016/j.ssresearch.2017.09.005

Tabernero‐Rico, P. M., & Garcia‐Velasco, J. A. (2019). Observational study of the social determinants of health in subfertile versus nonsubfertile women. *Journal of Human Reproductive Sciences, 12*(3), 240.

Weller, L., Weller, A., Koresh‐Kamin, H., & Ben‐Shoshan, R. (1999). Menstrual synchrony in a sample of working women. *Psychoneuroendocrinology, 24*(4), 449–459. doi: 10.1016/s0306‐4530(98)00092‐4

Zhu, J. L., Hjollund, N., Boggild, H., & Olsen, J. (2003). Shift work and subfecundity: A causal link or an artefact? *Occupational and Environmental Medicine, 60*(9), e12.

### B.4 Did not meet the criteria for outcomes (*n* = 2)

Dagher, R. K., McGovern, P. M., Dowd, B. E., & Lundberg, U. (2011). Postpartum depressive symptoms and the combined load of paid and unpaid work: A longitudinal analysis. *International Archives of Occupational Environmental Health*, *84*(7), 735–743. doi: 10.1007/s00420‐011‐0626‐7

Woods, N. F., Mitchell, E. S., Percival, D. B., & Smith‐DiJulio, K. (2009). Is the menopausal transition stressful? Observations of perceived stress from the Seattle Midlife Women's Health Study. *Menopause*, *16*(1), 90–97. doi: 10.1097/gme.0b013e31817ed261

### B.5 Did not meet the criteria for article type (*n* = 1)

Lynch, R., Lummaa, V., Panchanathan, K., Middleton, K., Rotkirch, A., Danielsbacka, M., O‧Brien D., & Loehr, J. (2019). Integration involves a trade‐off between fertility and status for World War II evacuees. *Nature Human Behavior, 3*(4), 337–345. doi: 10.1038/s41562‐019‐0542‐5
